# What do we know about who does and does not attend general health checks? Findings from a narrative scoping review

**DOI:** 10.1186/1471-2458-12-723

**Published:** 2012-08-31

**Authors:** Ruth Dryden, Brian Williams, Colin McCowan, Markus Themessl-Huber

**Affiliations:** 1 Social Dimensions of Health Institute, 11 Airlie Place, University of Dundee, Dundee, UK; 2 Nursing, Midwifery & Allied Health Professions Research Unit, Iris Murdoch Building, University of Stirling, Stirling, UK; 3 Division of Population Health Sciences, Ninewells Hospital & Medical School, University of Dundee, Dundee, UK

**Keywords:** Health checks, Screening, Public health, Inequalities, Primary care

## Abstract

**Background:**

General and preventive health checks are a key feature of contemporary policies of anticipatory care. Ensuring high and equitable uptake of such general health checks is essential to ensuring health gain and preventing health inequalities. This literature review explores the socio-demographic, clinical and social cognitive characteristics of those who do and do not engage with general health checks or preventive health checks for cardiovascular disease.

**Methods:**

An exploratory scoping study approach was employed. Databases searched included the British Nursing Index and Archive, Cumulative Index to Nursing and Allied Health Literature (CINAHL), Cochrane Database of Systematic Reviews (CDSR) and Database of Abstracts of Reviews of Effects (DARE), EMBASE, MEDLINE, PsycINFO and the Social Sciences Citation Index (SSCI). Titles and abstracts of 17463 papers were screened; 1171 papers were then independently assessed by two researchers. A review of full text was carried out by two of the authors resulting in 39 being included in the final review.

**Results:**

Those least likely to attend health checks were men on low incomes, low socio-economic status, unemployed or less well educated. In general, attenders were older than non-attenders. An individual’s marital status was found to affect attendance rates with non-attenders more likely to be single. In general, white individuals were more likely to engage with services than individuals from other ethnic backgrounds. Non-attenders had a greater proportion of cardiovascular risk factors than attenders, and smokers were less likely to attend than non-smokers. The relationship between health beliefs and health behaviours appeared complex. Non-attenders were shown to value health less strongly, have low self-efficacy, feel less in control of their health and be less likely to believe in the efficacy of health checks.

**Conclusion:**

Routine health check-ups appear to be taken up inequitably, with gender, age, socio-demographic status and ethnicity all associated with differential service use. Furthermore, non-attenders appeared to have greater clinical need or risk factors suggesting that differential uptake may lead to sub-optimal health gain and contribute to inequalities via the inverse care law. Appropriate service redesign and interventions to encourage increased uptake among these groups is required.

## Background

Anticipatory care [1] has increasingly been seen as a means by which the increasing demands of an aging population [2], growing numbers of people living with long term conditions, and persisting inequalities in health [3] may be addressed [4,5]. A key feature of such approaches are general and preventive health checks, defined as interventions which include a physical examination and/or an assessment of demographic and lifestyle risk factors which assess an individual’s current health or predict their chance of developing illness in the future [6]. These may be carried out for primary and secondary preventive purposes, as part of annual routine health check-ups required among older age groups [7], or embedded opportunistically within routine clinical encounters [8].

Regular community based general health check-ups are important for the early identification of risk factors for conditions such as heart disease, diabetes and stroke [9], as evidenced in the recent introduction of Health Check within the United Kingdom (UK) National Health Service (NHS). The evidence base to support such health checks rests predominantly on the known efficacy of the individual screening components subsumed within them. For example, recent National Institute for Health and Clinical Excellence (NICE) guidance on the prevention of cardiovascular disease points to the known effectiveness of interventions within health checks in relation to risk assessment, smoking, and physical activity [10]. Such preventive health strategies may therefore also provide a cost effective way of dealing with the causes of ill health before they manifest into serious long-term conditions.

Despite the potential importance and benefit of such health checks, their uptake is known to be largely sub-optimal [11]. For example, data illustrating the implementation of the recent NHS Health Check in the UK has shown uptake rates of around 50% [12]. Furthermore, there is good reason to think that the pattern of uptake is likely to be differentially spread across socio-economic groupings and thus follow the inverse care law [13,14]: those who have greatest to benefit from the services are least likely to engage with them. Differential uptake therefore has the potential to exacerbate health inequalities [14]. Consequently, knowledge of the socio-economic correlates of high and low uptake is important if current services are to be appropriately adapted in order to rectify such inequity.

Community based health-checks which aim to effectively and efficiently screen maximum proportions of eligible populations, are likely to be complex interventions consisting of numerous potential parameters: method of invite, location, timing, and nature (duration and content) of the screening process. Consequently, the development of new forms of health check should consider the theoretical and empirical basis to support maximal uptake [15,16].

This exploratory scoping study aims to establish the nature and extent of current knowledge relating to the uptake and engagement with general health checks and preventative health checks for the risk factors of cardiovascular disease in particular, and thus contribute to the development of such a theoretical and empirical basis to informal future service development. In particular, it sought to address three fundamental questions:

1. What are the socio-demographic characteristics of those who do and do not engage with health checks?

2. What are their stated reasons for not attending health checks?

3. What are the clinical needs and risk factors of these non-attenders?

## Methods

Establishing the state of knowledge with regard to a number of important but general questions requires a broad and inclusive review type rather than a highly focussed systematic review targeting a highly specified question around effectiveness. Scoping studies as defined by Arksey and O’Malley provide a structured but less restrictive alternative to the traditional systematic review of the literature [17]. They discuss four potential uses for a scoping study:

1. To examine the extent, range and nature of research activity

2. To determine the value of undertaking a full systematic review

3. To summarise and disseminate research findings

4. To identify research gaps in the existing literature” p6 [17].

This literature review followed an iterative scoping process which incorporated these objectives. The methodology was selected over the systematic review as its purpose was to explore the broad state of knowledge regarding attendance at general health checks rather than answer a clearly defined question. The breadth of potential studies and their heterogeneous nature meant that a scoping study with a narrative synthesis providing comprehensive representation of the evidence was more appropriate.

### Search strategy

A search of bibliographic databases did not identify any existing systematic review which focused specifically on this topic, and a decision was made to develop an alternative search strategy designed specifically for the project.

### Types of studies

This review considered both quantitative and qualitative studies including, but not limited to: project evaluations, randomised controlled trials, cohort studies, experimental or quasi-experimental trials, uncontrolled trials, systematic reviews, meta-analyses and studies using evaluation methodology such as the theory of change. Inclusion and exclusion criteria were developed using the ‘population, intervention, comparison and outcome’ (PICO) acronym as a framework [18], and are detailed in Table 1. Differences in the delivery of health care systems may mean that findings from studies in underdeveloped countries may not be relevant to the context of this project. This resulted in the decision to restrict studies to developed countries. Similarly, studies where health insurance was not controlled for were excluded from the review. Findings were restricted to papers on general or preventive health checks for the risk factors of cardiovascular disease, as other disease specific screening programmes (for example breast screening) have their own intricacies with barriers which are better understood and findings which are not always transferable. Papers on geriatric annual health checks were excluded as these were less likely to be of a preventive nature due to the age group and focused more on functionality and ability to live independently than clinical or lifestyle risk factors. Some papers which were retrieved considered general health checks and disease specific screening within the same study. Therefore, papers were included if they contained both disease specific AND general heart health checks, but excluded if disease specific (other than heart/cardiovascular disease) screening was the main focus of the paper.

**Table 1:** Inclusion & exclusion criteria

**Inclusion Criteria**	**Exclusion Criteria**
Population:	Population:
· Western/developed countries	· Children
· Hard to reach populations	Intervention:
· High risk groups	· Disease-specific health checks/screening (other than heart disease)
Intervention:	· Geriatric annual health checks
· General health checks	Control:
· Heart disease health checks	· Studies from the developing world
· General/Heart AND other disease-specific health check	Limits:
· Studies whose primary outcome was to increase uptake	· Non-English language papers
· Studies where uptake was documented (of the above interventions)	· Non-empirical opinion papers
Control:	· Papers published pre 1980
· Control group not necessary	
Outcome:	
· Initial uptake of screening and/or	
· Long term engagement with services	

### Databases used

The databases used for the review were the British Nursing Index and Archive, Cumulative Index to Nursing and Allied Health Literature (CINAHL), Cochrane Database of Systematic Reviews (CDSR) and Database of Abstracts of Reviews of Effects (DARE), EMBASE, MEDLINE, PsycINFO and the Social Science Citation Index (SSCI). A wide variety of databases were chosen to allow the complex concept of ‘uptake of services’ to be explored from a number of different disciplines. Searches were performed on each database individually to improve functionality and allow search terms and limits to be amended from the original template (Table 2) to meet each database’s specifications. Specific database search strategies and terms are available from the authors. Given that predictors of uptake are likely to change over time as cultures, values and services change, a judgement was made to exclude older studies. A subjective judgment was made to include papers published from 1996 onwards.

**Table 2:** Search strategy

**#**	**Search Term**
1	Health services for the aged
2	(MH “Health Promotion”)
3	(MH “Preventive Health Services”)
4	(MH “Primary Prevention”)
5	“health check”
6	“health examination”
7	“health examinations”
8	(MH “Family Practice”)
9	“general practice”
10	“opportunistic”
11	“health screening”
12	S1 or S2 or S3 or S4 or S5 or S6 or S7 or S8 or S9 or S10 or S11
13	(MH “Health Services Accessibility”)
14	(MH “Patient Acceptance of Health Care”)
15	(MH “Patient Dropouts”)
16	non-respon*
17	(poor attend* or non-attend*)
18	non-engage*
19	non-particip*
20	barrier*
21	(dropout* or drop* out*)
22	hard to reach
23	inverse care law
24	S13 or S14 or S15 or S16 or S17 or S18 or S19 or S20 or S21 or S22 or S23
25	S12 and S24
26	TI cancer or MW cancer or MJ cancer
27	S25 NOT S26
28	S25 NOT S26 (English language)
29	S25 NOT S26 (limited 1980–2010)

(*MH* exact subject heading, *MJ* word in major subject heading, *MW* word in subject heading, *TI* title).

### Selection process

The search and review procedure was conducted systematically and is outlined below with the initials of the researchers involved alongside:

• Ran search in databases individually (RD)

• Removed duplicates within databases (RD)

• Removed duplicates between databases (RD)

• Papers screened for relevance by title (RD)

• Papers independently screened for relevance by abstract (RD and CM)

• Meeting to discuss agreement (RD, CM, BW)

• Remaining papers screened using full text (RD and BW)

## Results

**Figure 1:**
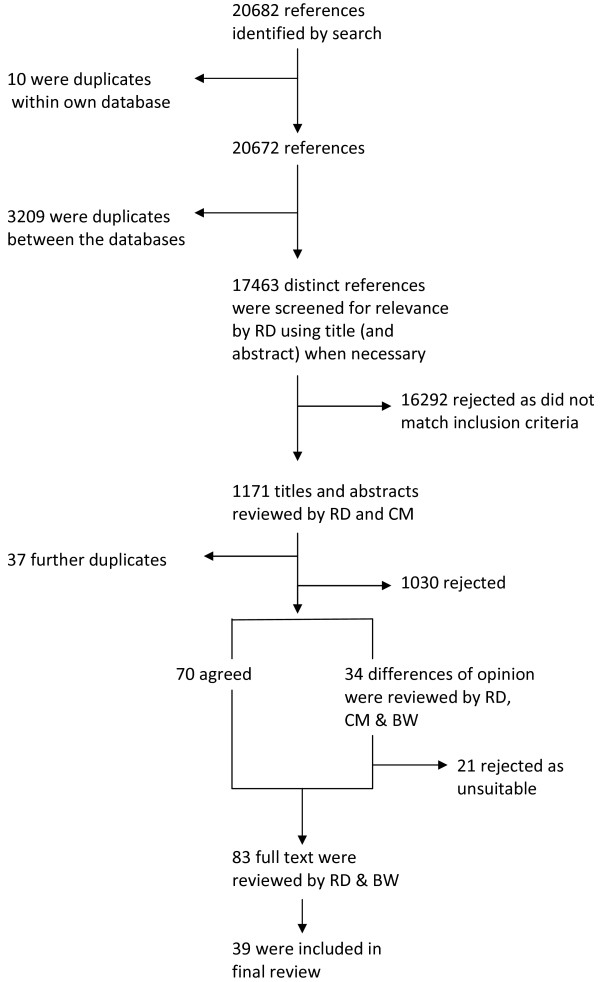
Flow diagram of review process.

A total of 17,463 studies were returned after searching the databases and performing electronic de-duplication within and between each database; the breakdown of papers by database is shown in Table 3 and the identification and exclusion of papers throughout the process is shown in Figure 1. A total of 39 papers were included in the final review (See Table 4). The findings of the literature review are presented below using a narrative synthesis reflecting the Economic and Social Research Council guidance [19].

**Table 3:** Hits by database

**Database**	**Number of references**	**Duplicates within own database**	**Distinct references**
Medline	8558	1	8557
CINAHL	3234	1	3233
BNI	148	0	148
SSCI	3902	1	3901
PsycINFO	1945	4	1941
EMBASE	2379	3	2376
CDSR + DARE	516	0	516
Total	20682	10	20672

**Table 4:** Summary of included studies

**Reference Number**	**Primary Author**	**Year**	**Title**	**Setting/Participants**	**Method**	**Key Findings**
20	Bletzer, K. V.	1989	Review of a health fair screening program in Mid-Michigan	America	Programme evaluation Evaluation of sociodemographic data on attenders at health fairs over seven years and findings from a survey with a sample of participants	· Women consistently outnumbered men by a ratio of at least 3:2 every year
				Health fair Open access 15124 participants		
						· Older people were more likely to present than younger people, with half of participants older than 50
						· 90% of those surveyed had consulted their GP within the past two years
						· The number of serious problems identified was low
						· The main reason for attendance was “curiosity about health”
21	Culica, D.	2002	Medical checkups: Who does not get them?	America	Telephone survey	· Reduced likelihood of having had health check in the previous 12 months was associated with being: 25-44 or over 65, male, unmarried, a smoker and in those who perceived cost barriers
						· Check ups were more likely in people who earned over $75,000, had health insurance, were physically active, had chronic disease and who rated their health as good, fair or poor rather than good or excellent
				Sample of 3600 individuals	Analysis of Iowa 1996 Behavioral Risk Factor Surveillance System	
22	Greenland, P.	2002	Attendance patterns and characteristics of participants in public cholesterol screening	America	Programme evaluation of cholesterol screening programme	· Participants more likely to be white (98.5% v 96.7%), older, female (59.9% v 51.6%) and better educated than the general population
				Cholesterol screening	Comparison of participant demographics with local census data	· 22% had previous diagnosis of high cholesterol and came to confirm/monitor previous readings
				Open access		· 79% came to the store specifically for screening
				10 supermarkets		· Time was an important factor as weekend and weeknights attracted more men and younger people than weekday screenings
				8583 people were seen over 4months		
						· Less than 5% took time off work to participate
23	Waller, D.	1990	Health checks in general practice: Another example of inverse care?	UK	Programme evaluation	· 1458 patients (65.9%) were offered screening
						· Of those invited 963 (66%) attended for a health check
				Attendance at General Practice health checks over	Medical record audit and postal questionnaire	· Attenders were more likely to be women, aged 45yrs or older, married, non-smokers and of higher social class than the non-responders to the invitation
				2211 men and women aged 35-64 were in the target age group		· Relative likelihood for non-attendance was 1.24 for smokers, 1.20 for the overweight, 1.16 for heavy drinkers, 1.28 for those with a less healthy diet
				Men were invited opportunistically, women were invited in the context of cervical smear tests		· Frequent GP consulters were more likely to attend
24	Jacobsen, B. K.	1992	The Nordland Health Study - Design of the Study, Description of the Population, Attendance and Questionnaire Response	Norway	Quasiexperimental and survey	· 82% attended the screening
				Health screening		· 78% men and 86% women attended
					Population screening and questionnaire	
						· Non-attenders tended to be single
						· 84% married men attended screening compared to 65% divorced/single or widowed men
				Letter invitation		
				10497 patients aged 40-42 were invited		· 88% married women attended compared to 79% divorced/single or widowed women
25	Simpson, W. M.	1997	Screening for risk factors for cardiovascular disease: A psychological perspective	UK	1. Quasi-experimental	· Overall uptake 62.4%; 59% at further education college, 28% at council cleansing department, 81% at greetings card factory.
				3 studies (only two were relevant to literature review)	Mobile screening programme and prospective questionnaire	
						· In general attenders were significantly older and more likely to be female than non-attenders
				1. Worksite screening at three workplaces:	2. Longitudinal	· Attenders were more likely to have had a definite intention to attend, and were more aware of the availability of the service
					Random allocation of invitation type	
				Further education college		· Non-attenders perceived more barriers to attendance and perceived themselves to be at higher risk of developing serious diseases
					Two questionnaires:	
				Council Cleansing department		
					One week after screening to assess intention to change behaviour	· The lower uptake at the council was attributed to the higher ratio of male to female employees, a lower education level and the youngest average age of all the workplaces
				Greetings card factory		
				Open access		
				2. Organisation of a screening programme		· Uptake varied by invitation type
					Three months after screening to measure behaviour change	· 100% opportunistic patients, 54% of those invited by letter and 29% personally invited attended the screening clinic
				General Practice		
				Uptake by invitation type:		· The method of offering screening did not affect changes in behaviour but those who engaged opportunistically were more likely to intend to smoke less.
				1. Opportunistic screening by GP		
				2. Invitation and fixed appointment to attend screening with practice nurse		· Patients who engaged after being invited by letter or personally were more likely to eat less fat and take more exercise than those who engaged opportunistically
						· Smokers were likely to attend than non-smokers
				3. Personal invite by GP to make appointment for screening clinic with practice nurse		
				210 male patients		
26	Thomas, K. J.	1993	Case against targeting long term non-attenders in general practice for a health check.	UK	Quasiexperimental Patient records were randomly sampled to assess attendance over a 3 year period.	· The median proportion of 3 year non-attenders was 23% in inner city practice compared to 9% in other practices
				30 General Practices		
				Mailed invitations		· 310/679 non-attenders were not contactable v 320/379 attenders who were contactable. This was related to last recorded consultation
				Random sample of 679 patients who had not attended for 3 years and 379 patients who had attended within this time		
					A sample of those who had attended in the past 3 years were invited for a health check and were invited to take part in a home interview two weeks before the health check	· Non-attenders were more likely to be female. Female non-attenders were more likely to be older than male non-attenders
						· Non-attenders scored significantly better on six measures of perceived health status and used less accident and emergency services and preventive health care than attenders
				Age 16-74		
					Non-attenders were invited to a health check but were not interviewed	
27	Wall, M.	2004	Non-participants in a preventive health examination for cardiovascular disease: characteristics, reasons for nonparticipation, and willingness to participate in the future	Sweden	Quasi-experimental	· 237 persons (76.7%) participated
				Ockelbo project	Preventive health examination	· Of 72 non-attenders at the health examination, 53 (73.6%) responded to the questionnaire, 14 (19.4%) agreed to a telephone interview and 5 (6.9%) did not respond
				309 persons aged 35 or 40yrs were invited to participate in a health examination		
					Follow up questionnaire mailed to nonparticipants	
					Follow up telephone interview with non-participants who did not respond to questionnaire	· The proportion of smokers was significantly higher in non-attenders v attenders at the health check (31.3% v 18.6%)
						· Reasons for non-attendance included: lack of time or hindrances at work (52%), already in contact with health services (33%), or because they felt healthy (21%)
						· However the majority of non-attenders (55%) said they would be interested in attending in the future, 28% said they were not sure, and 16% said they would not be interested
28	Cherrington, A.	2007	Do adults who believe in periodic health examinations receive more clinical preventive services?	America	Telephone survey	· Non-endorsers of periodic health examinations received less preventive services
				Telephone survey	Logistic regression analysis of phone survey to assess attitudes towards periodic health examinations and the receipt of preventive services	
				4879 respondents		· 8.5% (n=374) did not endorse annual periodic health examinations
						· Non-endorsers tended to be male (odd ratio (OR) 1.64), younger (OR 0.87), white (OR 2.91), to have at least some college education (OR 1.43) and feel healthy (1.85)
						· 56% of non-endorsers had received a cholesterol check in the previous 5 years compared to 81% of endorsers
29	Karwalajtys, T.	2005	A randomized trial of mail vs. telephone invitation to a community-based cardiovascular health awareness program for older family practice patients	Canada	Prospective	· 58.3% of invited patients attended
				1 family physician practice	randomised trial of invitation to attend community based by mail or telephone	· Patients invited by phone were more likely to attend than those by mail (72.3% v 44.0%)
				5 community pharmacies		· Patients with a family history of cardiovascular disease were significantly more likely to attend
				Telephone and mailed invitation		
				235 patients aged 65+	Health record review	
30	Hsu, H.Y.	2001	The relationships between health beliefs and utilization of free health examinations in older people living in a community setting in Taiwan	Taiwan	Cross-sectional survey	· Higher uptake of health examination in those with higher education and socio-economic status, and those with increased family support (6% of users lived alone compared to 13% of non-users)
				Free health examination in over 65s		
					Stratified random systematic sample of 200 men and women were given a 17 item health belief scale to complete	
				100 participants		· Users perceived a higher level of seriousness and susceptibility to ill health than non-users
				100 nonparticipants		
31	Bowden, R. G.	2001	Comparisons of cholesterol screening participants and non-participants in a university setting	America	Case–control analysis of participants in worksite screening	· Participants were more likely to be male (68.5% v 53.7%), older (47.0 years v 40.4 years), white (91.9% v 78.7%), have a college degree (85.9% v 51.3%) and have higher mean salaries than nonparticipants ($50,054 v $30,009)
				Worksite screening		
				University		
				Invite with pay check		
						· Barriers to uptake in non-attenders were suggested to be cost, less flexible working hours, lack of access to communication methods including email, conspiracy theories around the employer’s motives and that the workers did not feel sick and did not need screened
				270 participants		
				587 random sample of nonparticipants		
32	Franks, P.	1991	Barriers to Cholesterol Testing in a Rural- Community	America	Cross-sectional population based survey	· 24% reported prior cholesterol testing
				Cholesterol check		· Factors associated with a reduced likelihood of ever having a cholesterol test: age under 45, less than 12 years education, income of less than $10,00, no health insurance, no doctor visit in past year, practicing 3+ cardiovascular risk factors
				Invitation by telephone, leaflets and home visits		
					Logistic regression	
				557 households contacted 508 (91%) participated Survey of 1063 people		
				973 (92%) screened for cholesterol		
33	Jones, A.	1993	Comparison of risk factors for coronary heart disease among attenders and nonattenders at a screening programme	Wales	Case control	· Non-attenders were more likely to be older, have higher body mass index, cholesterol and blood pressure, and low socio-economic status, a personal/family history of heart disease, be smokers, have low educational level and high alcohol consumption than attenders
				General Practice	Random systematic sample of 1398 non-attenders identified 140 individuals who were repeatedly contacted and encouraged to attend a health check.	
				Mailed open invitation then fixed appointment mailed, telephone call and home visit for nonresponders		
						· Reasons given for not attending the initial screening programme were varied with 36.7% claiming not to have received the letter and 26.5% citing practical barriers
				3800 patients invited for health check		
				2402 (63.2%) attended		
					98 non-attenders eventually presented for a health check and their results were compared to initial attenders	
				Aged 25-55 years		
34	Sonne-Holm, S.	1989	Influence of fatness, intelligence, education and sociodemographic factors on response rate in a health survey	Denmark	Case control	· 964 obese (58%) and 1134 controls (75%) attended a health examination
				Health examination	Survey of cohort of severely obese men with a randomly selected control group invited to a health examination	
						· Regardless of study group, the response rate was independently associated with decreasing body mass index and increasing intelligence test score, educational level, social class, age up to 50 years old and proximity of residence to the screening location
				362,200 male draftees to Danish military board		
				Mailed invitation and reminder		
				1651 identified as severely obese draftees		
				1504 controls were randomly selected from the remaining population		
35	Walker, M.	1987	Non-participation and mortality in a prospective study of cardiovascular disease	UK	British Regional Heart Study	· 7735 men (74.3%) participated in the study
				Comparison of characteristics and mortality levels of participants and non-participants in clinical examination		· Non-participants had a significantly higher relative risk of death during the first three years after the screening date
					Prospective study of cardiovascular disease in middle aged men	
						· Non-participants were more likely to be younger, unmarried and less skilled workers than participants
				Sample of 10412 men aged 40-59 years		
36	Thorogood, M.	1993	Factors affecting response to an invitation to attend for a health check	UK	Quasi-experimental	· 2205 attended (82.3%)
				5 General Practices	Postal questionnaire before invite to attend a health check and subsequent record of attendance	· Non-attendance was higher in males than females (21% v 15%)
				Invitation by mail or telephone, or opportunistically plus up to 3 reminders		· Non-attenders were more likely to be single than married (24% v 16%), manual rather non-manual workers (21% v 15%), living in rented accommodation rather than homeowners (29% v 16%) and not have access to a car rather than be a car user (27% v 16%)
				2678 patients aged 35-64 were invited to attend a health check		
						· Non-attenders were less healthy than attenders as shown by following odd ratios: 1.74 smokers, 1.07 heavy drinkers, 1.91 less healthy diet, 1.50 for obese patients
						· Attenders were more likely to visit their GP frequently and indicate a willingness to change their behaviour
37	Dignan, M. B.	1995	Factors associated with participation in a preventive cardiology service by patients with coronary heart disease	America	Prospective cohort/Qualitative	· 24 patients (39%) attended the clinic
				Cardiology clinic		· No statistically significant demographic differences were found between attenders and non-attenders
				Face to face open invitation and follow up letter		
					Telephone interviews	· Patients who attributed their hospitalisation to a heart attack or coronary bypass surgery were more likely to attend the clinic than those who attributed admission to chest pain or for diagnostic reasons
				62 patients	Follow up of patients who were hospitalised for heart related conditions to assess reasons for nonattendance at secondary prevention clinic	
38	Griffiths, C.	1994	Registration health checks: Inverse care in the inner city?	UK	Survey	· Non-attenders were significantly more likely to be unemployed, African, heavy smokers and of lower social class than attenders.
				7 GP practices	Questionnaire analysis	
				Face to face open invitation		
						· Demonstrated that invitations to health checks given in an unselected way are least likely to engage with those in most need
				356 patients: 101 declined/provided inadequate data		
				Of the remaining 256 patients, 118 attended a health check (46%)		
39	Wilson, S.	1997	Health beliefs of blue collar workers: increasing self efficacy and removing barriers	USA	Cross-sectional, descriptive, expost facto questionnaire	· 151 (75.5%) completed questionnaires
				Health beliefs of participants and non-participants in worksite blood pressure and cholesterol screening		· 45 workers (22.5%) subsequently attended a health check
						· No significant difference between respondents and participants by age, race, education, gender, marital status, shift or health history
					Worksite screening	
						· Workers who participated in the screening had significantly higher self-efficacy and perceived significantly fewer barriers to participation than non-attenders
				Convenience sample 200 blue collar workers		
40	Boshuizen, H. C.	2006	Non-response in a survey of cardiovascular risk factors in the Dutch population: Determinants and resulting biases	Netherlands	Logistic regression of determinants of participation in a health examination survey in previous participants in a health interview study	· 28.9% patients participated in a health examination that had participated in an earlier health interview survey
				Health examination		
				3699 participants from a sample of		· Participants were more likely to be male and have high socio-economic status
				12786 previous participants		· Participation increased with age until 60 then decreased sharply thereafter
						· The rural population were less likely to participate
						· There was evidence of the “worried well” with frequent consulters and those with good health more likely to attend
						· The unemployed were least likely to attend but participation decreased with increasing hours of work
41	Pill, R.	1985	Invitation to attend a health check in a general practice setting: comparison of attenders and non-attenders	UK	Quasi-experimental	· Attenders were generally better educated, of higher social status, had greater health motivation, fewer ties and commitments, attended church more regularly, employed, performed more health approved practices, had had more recent contact with GP, and accepted the legitimacy of the doctor’s interest in their lifestyle than nonattenders
				Health check	Comparison of demographics, attitudes, beliefs, preventive health behaviour and past contact with the practice between attenders and non-attenders	
				General practice		
				Mailed invitation		
				Sample of 259 non-attenders and 216 attenders aged between 20 and 45		
						· Attenders were more likely to have no children under 5, no dependents and have fewer than 6 contacts a month with friends or relatives than nonattenders
						· Non-attendance was associated with greater perceived support from family and friends
42	Persson, L. G.	1994	A Study of Men Aged 33-42 in Habo, Sweden with Special Reference to Cardiovascular Risk-Factors	Sweden	Quasi-experimental	· 652 men (86.1%) had attended after one mail invitation
				Health check	Follow up of non-attenders by mailed questionnaire and telephone	
				Postal invitation plus two reminders		· Of 105 non-participants, 16 were known high consumers of health care, 40 had recently had a health examination (mostly at work) and 49 were not interested in a health check
				757 men aged 33-42 were invited to attend for a health check		
						· Non-attenders were more likely to be single, smokers, on the sick list, on a lower income or more often unemployed than attenders
43	Christensen, B.	1995	Characteristics of attenders and non-attenders at health examinations for ischaemic heart disease in general practice	Denmark	Quasi-experimental study	· Attendance was higher in free health examinations than those which charged a fee (66% v 37%) · Attendance was significantly lower in single men than cohabitants
				65 General Practices	Multi-practice study and questionnaires to assess the influence of a fee to attend a health examination	
				Health examinations for ischaemic heart disease		· Whether the service was free or not was the biggest predictor of attendance as health beliefs of attenders and non-attenders were similar
				Letter invitation 2452 men aged 40-49 years were invited to attend		
44	Difford, F.	1987	Continuous opportunistic and systematic screening for hypertension with computer help: Analysis of nonresponders	England	Programme evaluation	· 2354 patients (92%) had blood pressure recorded in the previous 5 years after 2 years
				General practice	Audit of medical records	
				Opportunistic hypertension screening		· Those who had been screened have higher consultation rates (6x greater) than non-responders
					Analysis of characteristics of 192 nonresponders	
						· There was no difference by distance to the practice or number of years registered with the practice
				2546 patients aged 40-64 years		
						· The only significant difference was that nonresponders were the only people in a household registered with a practice which was interpreted that they were either single or had a lack of need to identify with the “family” doctor
45	Engebretson,J.	2005	Participation in Community Health Screenings: A Qualitative Evaluation	America	Qualitative Focus groups	· Described domains of motivation for presentation:
				Participants in screenings at 5 settings:	5 with attenders	· Self-care orientations (e.g. self-assessment/no perceived need)
					1 with nonattenders	· Interpersonal influences (e.g. endorsement by others/fear of embarrassment)
				University employees		
						· Accessibility (e.g. convenience/lack of time)
				County fair attendees		· Overlap of facilitators and barriers to participation; what motivated one participant to attend may act as a barrier to another
				Senior citizen centre clientele		
				Local industry employees		
				University student		
				Group of non-attenders		
46	Harpole, L.H.	2000	Feasibility of a tailored intervention to improve preventive care use in women	America	Survey to identify outstanding preventive health care needs	· 591 women (67%) returned the survey
				Survey mailed to 893 women aged 50-55		· 76% were in need of one or more preventive health service
						· 16% were in need of 3 or more
						· Women with increasing need for preventive health services were more likely to be non-white, earn less, have a lower level of education, and be less satisfied with their health care
47	Norman, P.	1991	Predicting attendance at health screening: Organizational factors and patients’ health beliefs	UK	Programme evaluation	· 131 (59.3%) questionnaires were returned. From this group 98 attended and 33 did not attend the subsequent health check
				General Practice	A health belief questionnaire was sent to sample of 221 patients who were subsequently invited for screening	
				Health check		
				Mailed fixed appointment or invited opportunistically		· The two invite methods had similar attendance rates but the letter invite was more efficient, as opportunistic screening relied on patients presenting at their GP before they could be invited
				325 patients aged between 30 and 50		· Opportunistic screening was slightly biased in favour of females
						· Attenders were more likely to report cutting back on daily activities when ill and believe in the seriousness of high blood pressure and weight problems
				Health belief questionnaire	11 patients were interviewed directly after their screening appointment	
						· Non-attenders were found to be more worried about the screening appointment and perceived more barriers to attendance
48	Shiloh, S.	1997	Correlates of health screening utilization: The roles of health beliefs and selfregulation motivation	A convenience sample of 252 asymptomatic individuals were invited to participate in one of four screening programmes: dental check up, blood pressure measurement and cholesterol testing, pap smear or mammography	Quasi-experimental	· 137 (54%) attended and 115 (46%) did not attend
					Analysis of participants in a screening programme	· Motivations and health beliefs varied by screening programme
						· Non-attenders were more likely to justify their nonattendance behaviour with danger control motivations than fear control ones
					Questionnaire tailored to specific screening programme and whether individual attended or did not attend	
						· 61% non-attenders did not believe in the efficacy of screening in reducing their illness threat whereas 39% were too afraid of the possible results to attend
49	Norman, P.	1993	The role of social cognition models in predicting attendance at health checks	UK	Prospective survey/programme evaluation	· 419 patients were sent open invitations
				General Practice		· 399 patients were sent fixed appointments
				Mailed invitation with fixed appointment time or open invitation		· 433/818 patients attended a health check; 69.7% of those sent fixed appointments and 37.1% sent open invitations attended
					Health belief questionnaires sent before patients received invite letters	
						· Questionnaire data showed that for those that were sent a fixed appointment, attenders were more likely to place a high value on health, to believe health is influenced by powerful others, to be advised by referent groups to attend, to believe in the positive outcomes of screening and to not be affected by motivational barriers than nonattenders
				818 patients aged between 30 and 41 were invited to attend a health check		
					Health check	
					Patients randomly allocated to receive either a letter of invitation with either a fixed appointment or an open invitation to make their own appointment	
						· For those sent an open invitation, intention to attend and perceived control were independent predictors of attendance behaviour
50	Norman, P.	1991	Patients’ views on health screening in general practice	UK	Programme evaluation	· Of the 168 invited by letter, 121 patients (72%) attended a health check
				General Practice		
				Mailed fixed appointment or invited opportunistically	Patients randomly selected to be invited to general health screening in one of two ways:	· Only 83/157 patients had been invited opportunistically, but attendance in those that had been invited was 74.7%
						· The remaining patients who had not yet been invited opportunistically were sent a fixed appointment which produced 55.4% attendance
				Sample of 379 patients aged 30- 50 years, 325 were invited after exclusion of unsuitable patients		
						· 159/224 patients returned their questionnaires
					Letter with fixed appointment (n=168) or notes were tagged so patient was invited opportunistically to make an appointment for a health check when they presented at the practice for another reason (n=157)	· Those invited opportunistically were most likely to report that keeping their appointment time was easy, and were least likely to change it.
						· Those given fixed appointments experienced more difficulty in attending even if they were well motivated
					Questionnaire was issued after health check to assess views of health check	
					11 patients were interviewed	
51	Nielsen, K. D. B.	2004	“You can’t prevent everything anyway”: A qualitative study of beliefs and attitudes about refusing health screening in general practice	Denmark	Qualitative	· Reasons for non-attendance: too busy, healthy, recent contact with general practice, don’t want to know if ill, no symptoms, major life events, actual health problems
				Health examination	Interview with sample of 18 non-participants in a randomised control populationbased project	
				6 men		
				12 women		· They stressed the importance of autonomy, and that they would go to see their doctor when they needed to
					Non-participants were sampled using stratified purposeful techniques	
52	Norman, P.	1989	Intention to attend a health screening appointment: Some implications for general practice	UK	Cross-sectional survey	· Initial questionnaires were returned by 178 patients (37% response rate)
				General Practice	Patients randomly selected from practice list by age/sex bands (25-30, 35-40, 45-50 years)	
				Questionnaire to assess predictors of intention to attend a health check		· Reminder questionnaire returned a further 97 replies. An additional 29 questionnaires were excluded due to incorrect addresses or being incompletely filled in. Response rate was 57% (n=275)
				479 patients aged25-50		· Those who intend to attend a health check placed a high value on their health; believe in their susceptibility to common illnesses and the severity of major illnesses. They believe in the efficacy of doctors and screening, have someone to talk to about problems and are more likely to be married or cohabiting.
					Sent questionnaire	
						· Those who are likely to not attend have different attitudes towards screening and believe it would be too much effort or feel concerned about aspects of screening
53	Williams, A	2001	Cultural sensitivity and day care workers: examination of a worksite based cardiovascular disease prevention project	USA	Programme evaluation of screening initiative over three years	· Participation rates were increased from 26% to 73% over the duration of the project by adapting recruitment strategies to the target group’s cultural values and lifestyles, and building trust
				“Healthier people health risk appraisal”		
				Strategy to recruit child day care workers in a cardiovascular disease screening and risk reduction programme		· 70% of participants cited convenience (because it was offered at their workplace) and the fact that it was free as motivators to attend
					Interview with participants	
						· A lack of knowledge of cardiovascular risk was identified in this population as just over 10% of participants were aware of their blood pressure or blood cholesterol
				N=84		· Non-participants had been tested recently or were not interested in the screening at the time it was offered
54	Ornstein, S. M	1993	Barriers to adherence to preventive services reminder letters: the patient’s perspective	USA	Qualitative Telephone survey (n=307)	· 307 patients were surveyed by telephone to assess reasons for non-response to a letter for screening
				Cholesterol screening		
				Reminder letters sent to 1077 patients	Focus groups of non-responders to a reminder letter (n=27)	· 154 (50.2%) did not recall receiving the letter, 84 (27.4%) recalled receiving the letter but not its content, 69 (22.5%) recalled both
						· Highlighted the importance of the format and content of reminder letters to improve uptake of cholesterol checks by making them distinguishable from a bill, conveying a personalised message and addressing logistical barriers
55	Pill, R.	1988	Invitation to attend a health check in a general practice setting: the views of a cohort of nonattenders	UK	Qualitative	· 236 (91%) recalled getting the invitation, 3% could not remember and 6% denied ever receiving the invitation
				259 men and women aged 20- 45 who did not respond to a mailed invitation for a health check at General Practice	Interview of nonattenders	
						· Reasons for non-attendance: 44% were not interested, 24% forgot to attend, 26% cited crises at home or work, 11% felt screening was inappropriate
56	Thompson, N. F.	1990	Inviting infrequent attenders to attend for a health check: costs and benefits	UK	Quasi-experimental	· 17/94 patients (18%) attended
				General Practice	Audit of sample of practice records (n=1488) to identify all 3- year nonattenders (n=114) an invitation including fixed appointment time was sent to 94 eligible patients	· Of the remaining 77 patients, 3 had moved home, 28 cancelled the appointment and nothing was heard from 45, the final patient had been admitted for a myocardial infarction before the appointment
				Mailed fixed appointment		
				94 patients who had not attended general practice within the previous 3 years were invited for a health check		
						· Of those who cancelled, 8 were working or studying away from home, 4 found the appointment time unsuitable but did not wish to rearrange and 16 did not need or want an appointment
						· Those presenting were in general healthy with low levels of smoking and alcohol consumption and mild hypertension only diagnosed in one patient.
57	Hegarty, V.	1995	Reasons for nonresponse among older adults	UK	Letter to the editor describing study which invited over 75s for a health check	· 847 attended
				General practice		· 182 were untraceable (had moved home or were deceased)
				1342 invited for a health check		
						· 44 actively declined
						· 142 attended after a follow up telephone call
						· 120 did not attend because they had seen their GP within last 12 months
					Reasons for nonresponse were assessed with a questionnaire	
						· 7 did not respond because of ill health
						· The variety of reasons for non-response indicated that non-attendance does not always equate to poor health
58	Levine, J. A.	1991	Are patients in favour of general health screening?	UK	Cross-sectional survey	· 315/375 (84%) attenders completed the questionnaire
				General Practice	Questionnaire	
				375 consecutive patients 198 individuals who had not attended general practice for 12 months		· 93/198 (47%) non-attenders completed the questionnaire · A significantly greater proportion of attenders (83%) indicated they would make an appointment and attend for health screening compared to nonattenders (66%)
						· 33% of attenders would seek health screening even if not contacted by their doctor v 16% of nonattenders

### What are the socio-demographic characteristics of those who do and do not engage with health checks?

Studies consistently indicate that males are less likely to engage with health checks or screening and to endorse periodic health examinations than females [20-28]. This difference in rates of non-attendance between males and females ranged from 8% to 19% in those invited for a health check at General Practice [21,23,24]. In community based drop-in sessions, women were more likely to self-present than males, with the proportion of attenders at least 60-65% female [20,22]. Additionally, 11% of men compared to 6% of women did not endorse periodic health examinations [28]. Two other studies found no difference in attendance rates by gender [29,30].

In general, attenders at health checks are older than non-attenders [20-23,25,31-35], although some studies found no association between age and attendance [29,30,36-39]. In many cases the demographics of engagers were dependent on the targeting strategy of the intervention; for example where the service was only offered to a particular age group. Some of the included studies were targeted specifically at older adults while others were offered to an entire adult practice population. Although there was a tendency for attenders to be older than non-attenders, the heterogeneous nature of the study methodologies meant that it was difficult to define an optimum age for uptake. Indeed, the relationship between age and participation may not be linear. For example, participation in a health examination after completion of a health interview in the Netherlands followed a curve which rose with increasing age until 60 then declined significantly with any age above this [40].

Individuals were found to be less likely to attend if they had low socio-economic status [23,33,34,36,38,40,41]. Defining which socio-economic/demographic characteristics differentiate between attenders and non-attenders was complicated by the numerous ways social status was reported in the literature. Some studies discussed social class, employment status, occupational training and level of education or years spent in education independently; whilst others used the terms interchangeably or as proxy measures for each other. In general lower uptake was associated with low incomes [21,30-32,42], being unemployed [38,41,42] and lower educational attainment [22,25,27,31-34,41]. Although these terms may be closely related, one study found that each had an independent effect on the attendance rate [34].

An individual’s marital status was found to affect attendance rates with non-attenders more likely to be single [21,23,24,35,36,42]. Studies suggested a possible interaction between marital status and gender in explaining uptake. For example, a number of studies reported that attendance at health checks was higher in males who were married or cohabiting, compared to single males [21,24,35,42-44]. A possible explanation was proposed in a qualitative study using focus groups with participants and non-participants in community health screenings, which found that the decision to attend a screening is often made by the partner, with this initiation behaviour prevalent across a number of socio-demographic factors [45].

The tendency of women to present more than men (as evidenced earlier) persists regardless of marital status. Higher rates of attendance in women who were single, divorced or widowed (79%) were found compared to men with equivalent marital status (65%). Furthermore, the rates of attendance were 88% in married women and 84% in married men, indicating that being married appears to have a stronger effect on uptake in men [24]. Other studies have found no relationship between marital status and attendance rates [39,41,46].

In general, white individuals were more likely to engage with preventive health services than individuals from other ethnic backgrounds [22,31,38,40,46]. However, ethnicity was only reported in a small proportion of the studies (Seven of 39 papers). Only one of these reported no difference according to race [39]. One paper reported a higher proportion of non-attenders at registration health checks were of African origin [38]. On the other hand, a large American survey (n = 4879) found that 9.6% of white people did not believe in periodic health examinations compared to 3.4% of black people, and that black people were more likely to have been screened for cholesterol in the past 5 years than white people [28].

### What are patients' reasons for not attending preventative health checks?

The relationship between social cognitive factors and attendance behaviour was not straightforward as although health beliefs were found to affect uptake [47], the factors influencing the decisions of attenders and non-attenders may not necessarily reflect “opposite motivations of beliefs” [48]. To clarify, this meant that attenders may present for screening to reduce the fear or perceived danger of a condition, while non-attenders may have used the same rationale to not present, e.g. they did not feel at risk or were too frightened of the possible outcome if they did attend.

Despite this caveat, non-attenders were shown to value health less strongly, have lower self-efficacy, feel less in control of their health and be less likely to believe in the efficacy of screening [39,49]. Components of the health belief model were identified as significant predictors, with those who did not engage with services less likely to feel susceptible to ill health or perceive the conditions being screened for as serious as those who attended [25,30,48].

Individuals may present in response to symptoms, a family history of the condition [29], or to seek reassurance [50]. Others are simply interested in their health, seek confirmation of a previous reading/monitor an existing condition or are worried well [20,22,45]. Those who do not present may have no perceived need for a health check: they may feel healthy or have an absence of symptoms [27,33,51,52], are already in contact with the health service [27,33,41,51,53], or have recently had a health check [27,51,54]. Alternatively, they are aware they are unhealthy and do not want to be told off and have to make lifestyle changes, or have a fear of what it might find [26,28,31,47,51].

Fear acted as a barrier to uptake of screening in a number of ways, including: a fear of what the health check might find [33,52,55], the belief that “what I don’t know won’t hurt me” [45] and that knowing wouldn’t make them any happier [51], or the fear of the test results [25,45] and their consequences [51]. Concerns related to the procedure itself were also apparent in relation to a fear of needles or a general fear of doctors or medical settings, anxiety about what the tests might involve [45,47] or the experience level of those carrying out the tests [45].

### What are the clinical needs and risk factors of those who present for health checks?

Non-attenders had a greater proportion of cardiovascular risk factors than attenders. Smokers were less likely to attend than non-smokers [21,23,25,27,32,33,36,38,42,46,56]. Unhealthy lifestyle factors were important predictors of non-attendance, with odds ratios higher for smokers, heavy drinkers, and those with unhealthy diets and the obese [36]. However, one paper showed occasional smokers and ex-smokers were more likely to receive a check-up than non-smokers [21] and smokers with the intention of giving up were more likely to attend than those who did not want to [23].

A personal history but not family history of coronary heart disease (CHD) was significantly more common in non-attenders, as was a higher body mass index (BMI) [34], high cholesterol, systolic and diastolic blood pressure values [33].

Follow up of non-participants in a prospective study of cardiovascular disease found that this group were more likely to have died than participants in the three years following the health checks. The difference in the mortality rates between participants and non-participants was biggest in the youngest age group (40–44 year olds), indicating premature death. However, the mortality rates were not significantly different between these groups for cardiovascular disease or cancer [35].

The vast majority of studies supported the higher risk profile of non-attenders; however, non-attenders were found to have lower levels of cholesterol than those who did attend in a post-study follow up [32]. In another study initial responders had higher total cholesterol but lower diastolic blood pressure than those who had to be re-contacted [24].

In general, frequent or recent consulters at General Practice were found to be more likely to present for a health check [23,32,36,40,41,44] but for some people this recent or on-going contact can be a reason not to attend [27,42,51,57]. Consequently, this inconsistent relationship between frequency of attendance at GP practice and the likelihood of participation in preventive health screening has been described as ‘complex’ [40], and the two areas are inherently related. Some studies have shown that frequent or recent GP consulters are more likely to attend for a health check [23,32,36,40,41,44]; for example, over 90% of patients who attended a health check at a shopping centre reported having a regular source of medical care [22]. Conversely, other patients cited recent or ongoing contact with their GP as reasons for not attending a health check [27,42,51,57]. A survey of attenders and non-attenders at General Practice in the past year showed that attenders were more likely to indicate that they would make an appointment for a health check compared to non-attenders (83% v 66%) [58].

## Discussion

This review identified a substantial number of primary empirical studies contributing data to questions of uptake. Although the heterogeneous nature of interventions and populations precluded formal statistical meta-analysis, there appeared sufficient commonality across studies to inform a number of key conclusions. Routine health check-ups appear to be taken up inequitably with gender, age, socio-demographic status and ethnicity all associated with differential service use. Furthermore, non-attenders appeared to have greater clinical need or risk factors suggesting that differential uptake may lead to sub-optimal health gain and contribute to inequalities via the inverse care law. Our findings provide an initial contribution to the development of programme theories or conceptual frameworks to underpin future strategies, as suggested by NICE and others [59,60].

### Limitations of the review

Established and appropriate search strings were not available thus necessitating the development of new strategies. Like all reviews we cannot guarantee that studies have not been missed. However, our emphasis was on sensitivity over specificity resulting in almost 18,000 papers being examined by members of the team. We therefore believe that it is likely that few papers were missed. The purpose of the review was to identify sufficient studies across diverse contexts to inform the theoretical and practical development of future interventions to improve uptake of health checks. This necessary focus on diversity also meant that formal statistical meta-analysis or meta-regression of predictors of uptake would have been inappropriate.

The majority of studies came from North America (n = 13) and Europe (n = 24), and the remaining two papers were from Israel and Taiwan. There may have been benefits from loosening inclusion criteria to include both geriatric health checks and non-developed countries. Such diversity could potentially lead to sufficient numbers of papers with common interventions or populations as to justify a number of meta-analyses of effectiveness or meta-regression of predictors of uptake. While the scoping nature of this study precluded such an approach for pragmatic reasons we have demonstrated that such a review may be feasible and desirable in the future.

### The inverse care law in operation

In his original description of the inverse care law Julian Tudor Hart’s argued that “the availability of good medical care tends to vary inversely with the need for the population served” [14]. The validity of his law was demonstrated in a number of studies and in a number of ways in our review. Men from lower socio-economic backgrounds and on low incomes were consistently found to be less likely to engage with check-ups than women or people from a higher socio-economic status. Both of these variables are well established risk factors for a range of clinical conditions, perhaps most importantly in the context of this study, cardiovascular disease. This, again, was reinforced through this review since non-attenders were consistently found to have a range of poor lifestyle behaviours including smoking, alcohol consumption and diet. These findings suggest that without adaptation or increased efforts to increase uptake from these more “needy” populations there is the possibility that health checks, like other contemporary public health policies, risk exacerbating rather than narrowing health inequalities [61].

### Implications for future service design

Given the diversity of populations, clinical needs and motives not to attend health checks, a “one size fits all” solution consisting of promoting attendance at health checks and subsequent support for behaviour change is simplistic and flawed, particularly in the interaction with patients with complex needs [62]. Indeed, the current focus on a limited number of delivery types, and a failure to tailor services may at least in part contribute to the problem. However, while it would appear sensible to assume that complex problems require complex solutions, there may be exceptions. The increasing role of both social marketing and financial incentives as drivers of behaviour change both focus on increasing perceived value while not essentially changing the service itself or addressing many of the pre-stated barriers. Incentive based schemes are gaining significant attention as a means of promoting behaviour change through extrinsic motivations [63-65]. However, such schemes have led to a number of questions with regard to political acceptability, ethical justification and effectiveness. In addition, questions over their ability to sustain behaviour modification, once an incentive is withdrawn, were raised [64]. Given the preponderance of people on lower incomes among non-attenders, incentive schemes, whether based on finance or benefits in kind, may prove particularly effective and could be considered.

If tailoring of health check-ups is to take place then consideration would need to be given to the varied demands that this would place on health professionals charged with delivering the service. Among the challenges surrounding service delivery are clinician’s frequently low adherence to protocols on prevention within consultations [66-68]. This may be related to a lack of awareness of, and agreement with, guidelines, or a belief that many practices and outcomes would be difficult to change due to time pressure and other issues [69,70].

Moreover, clinicians in deprived communities are faced with higher rates of ill health and multi-morbidities, poor patient access, and high stress levels among clinicians, which in turn lead to higher demands on the service and service provider [71]. Diversifying the provision of health checks to multiple tailored forms may well exacerbate these pressures and reduce service compliance to such new protocols unless tailoring is largely cost and time neutral. Certainly, increasing intervention complexity may be associated with reduced levels of implementation. An alternative approach may be to provide increased emphasis on opportunistic health checks at routine consultations; although even this has still been found to be time consuming [8]. However, it has recently been suggested from a substantive evaluation of a complex outreach prevention service that the complexity of reasons for non-engagement among some people may not be predictable or “read in advance” [59]; this would suggest that whatever tailoring to services is made there will always be an imperative on the skill of the clinician to judge and respond to unique opportunities within the opportunistic consultation as well as wider systems approaches [59].

## Conclusion

All of these challenges and complexities indicate that a diverse range of approaches may be required if the full benefit of health checks are to be realised. While tailoring and targeting the form of delivery may have a role to play, it is likely that their implementation would require increased investment to ensure adoption and sustainability, particularly if narrowing health inequalities is a serious and central goal of such health checks. The Marmot report “Fairer Society Fairer Lives”, recently argued for a policy of “proportionate universalism”:

“Focusing solely on the most disadvantaged will not reduce health inequalities sufficiently. To reduce the steepness of the social gradient in health, actions must be universal, but with a scale and intensity that is proportionate to the level of disadvantage. We call this proportionate universalism.” p15 [72].

Such proportionate universalism would define “tailoring” as much in terms of the scale and intensity of action required for those most in need, as much as any changes in objective intervention form. Whatever approach is adopted, it is important that a clear theoretical underpinning that acknowledges both the complexity of the diverse population needs/attitudes and the challenges currently facing primary care and associated public health services is required. This synthesis of current findings has attempted to make a contribution to such a development.

## Competing interests

The authors declare that they have no competing interests.

## Authors’ contributions

RD designed the study, conducted the review and contributed to the writing of the paper. BW was involved in the design of the study and wrote the paper. CMC contributed to the review and the paper. MTH contributed to the review and the paper. All authors read and approved the final manuscript.

## Pre-publication history

The pre-publication history for this paper can be accessed here:

http://www.biomedcentral.com/1471-2458/12/723/prepub
